# A voltammetric determination of caffeic acid in red wines based on the nitrogen doped carbon modified glassy carbon electrode

**DOI:** 10.1038/srep45924

**Published:** 2017-04-05

**Authors:** Natarajan Karikalan, Raj Karthik, Shen-Ming Chen, Hsi-An Chen

**Affiliations:** 1 Electroanalysis and Bioelectrochemistry Lab, Department of Chemical Engineering and Biotechnology, National Taipei University of Technology, No.1, Section 3, Chung-Hsiao East Road, Taipei 106, Taiwan (R.O.C)

## Abstract

We reported an electrochemical determination of caffeic acid (CA) based on the nitrogen doped carbon (NDC). The described sensor material was prepared by the flame synthesis method, which gave an excellent platform for the synthesis of carbon nanomaterials with the hetero atom dopant. The synthesized material was confirmed by various physical characterizations and it was further characterized by different electrochemical experiments. The NDC modified glassy carbon electrode (NDC/GCE) shows the superior electrocatalytic performance towards the determination of CA with the wide linear concentration range from 0.01 to 350 μM. It achieves the lowest detection limit of 0.0024 μM and the limit of quantification of 0.004 μM. The NDC/GCE-CA sensor reveals the good selectivity, stability, sensitivity and reproducibility which endorsed that the NDC is promising electrode for the determination of CA. In addition, NDC modified electrode is applied to the determination of CA in red wines and acquired good results.

Caffeic acid (CA, 3, 4-dihydroxycinnamic acid) is one of the most important compound in the classification of phenolic acids. CA is found in red wines, cloves, coffee, star anise, olive oil and some vegetables and fruits^1^. It has several pharmacological functions, such as antioxidant, anti-inflammatory, antibacterial and immunemodulatory^2^,^3^,^4^,^5^. The two hydroxyl groups of CA significantly contributed to the unique antioxidant properties^6^. Interestingly, some studies reported that CA acts as an anti-tumor agent, however, some reports showed that CA has carcinogenic effects^7^. Thus, the quantitative detection of CA attains great significance to comprehend our daily diet. Moreover, the determination of CA in wines received a considerable importance in quality control analysis. Hitherto, various analytical methods have been developed to determine the CA, including capillary gas chromatographic method^8^, liquid chromatography–electro spray ionization mass spectrometry^9^, high pressure liquid chromatography (HPLC)^10^,^11^, capillary electrophoresis^12^ and voltammetric method^13^. These reported methods need sophisticated facilities and highly skilled technicians to operate those instruments, furthermore, they are time consuming process. Among all, the electrochemical techniques are cost effective, sensitive, selective and rapidly determined the CA. Several electrochemical CA sensors were developed based on the various modified electrodes. Particularly, Sousa *et al*. reported a CA sensor by the activated glassy carbon electrode (GCE), additionally, they have investigated the other phenolic antioxidants in orange juice^14^. Followed by them, Fernandes *et al*. reported a CA sensor based on the green beans homogenate and determine the CA in white wine^15^. Accompanied with those reports, Santos *et al*. developed a poly (glutamic acid) film modified CA sensor^16^ and LF da Silva *et al*. studied the properties of glassy polymeric electrodes modified with poly (caffeic acid) films towards the CA determination^17^. In addition, the lead modified GCE (PbFE-GCE), laccase-MWCNT and gold nanoparticles modified electrodes were reported for the electrochemical determination of CA^18^,^19^,^20^. Recently, the chemically reduced and electrochemically reduced graphene oxide modified electrodes are investigated for the determination of CA which revealed a superior performance^21^,^22^. Commonly, graphene or derivatives of graphene have been widely used in many different applications due to its remarkable physicochemical properties^23^. However, the bulk synthesis of graphene has to be improved from its limited synthesis methodology which includes thermal exfoliation or mechanical exfoliation of graphite, chemical vapor deposition on silicon wafers and the reduction of graphene oxide (GO)^24^,^25^,^26^. The Hummers method provides the foremost platform to the synthesis of GO, however, it may be exploded due to the chemical oxidants. Hence, the recent researchers concentrated on the various forms of carbon nanomaterials which expose high surface area and acceptable properties as similar to graphene. Moreover, the simple methodologies and preparation of carbon with hetero atoms dopant attains great interest. Commonly, the hetero atom doped carbon materials are exhibiting the superior electrochemical performance compared to undoped carbons. The current researches focused on the nitrogen, sulfur and phosphorous doped carbon for the electrochemical energy devices and sensors. The loan pairs on the hetero atoms were changed the physicochemical properties of the carbon lattice. Moreover, the hetero atoms shuttles the electrons between the orbitals of carbon and hetero atoms. Hence, we concentrate on the preparation of nitrogen doped carbon (NDC) by the burning of pyrrole. We have slightly modified our previous methodology, to improve the N content in carbon lattice^27^. The prepared compound was confirmed by various physical characterizations and applied to electrochemical determination CA.

## Results and Discussion

### Choice of materials

To date, graphene and graphene derivatives are dominating the realm of sensors and other applications due to their versatile physicochemical properties. Besides graphene, the carbon nanotubes and fullerenes are employed in the determination of biological compounds. However, aforementioned compounds are highly prestigious and synthesized by high equipped instrumental facilities. Nowadays, graphene can be synthesized by Hummer’s method, but, it has some limitations with the safety issues. Hence, we have chosen the simple carbon material especially with hetero atom dopant which synthesized by simple flame synthesis method. We believe that, this method will be an alternative route to prepare carbonaceous materials with hetero atom dopant (such as sulfur, nitrogen and phosphorous). Herein, we have described the electrochemical determination of CA by using NDC and achieved good analytical results. The obtained results are excellent when compared with previously reported carbonaceous materials and metal composites.

### Characterizations of NDC

**Figure 1:**
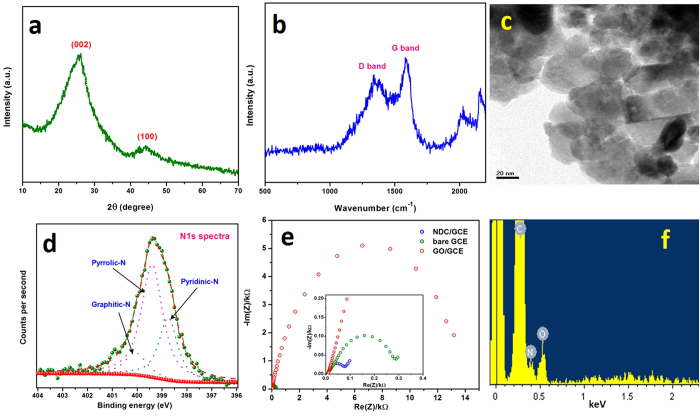
XRD pattern (**a**), Raman spectrum (**b**), TEM image (**c**) high resolution N1s spectrum (**d**), EIS (**e**) and EDAX spectra (**f**) of NDC.

The X-ray diffraction pattern of NDC was shown in Fig. 1a, which exhibited a predominant peak at 25.91° (2θ) and minor broad peak at 44.71°. These peaks are attributed to the (002) and (100) plane, further, the predominant peak shows the interlayer spacing of 0.342 nm along with the *c-*axis. The observed value is strongly matched with the pristine graphite which has the interlayer spacing about 0.34 nm. Nevertheless, the pristine graphite was revealed a strong and sharp peak at 26.5° because of its highly oriented carbon lattice^28^. The XRD results signified that NDC has the graphitic nature and crystallized in the hexagonal ordered unit cell. However, the broad and noised peaks indicated that the NDC has some structural defects and disorder in its crystal lattice. This behavior was arisen from the presence of nitrogen and oxygen moieties.

The Raman spectroscopy is highly satisfactory tool to examine the disorders of carbon nanomaterials, hence, the NDC was intrigued by Raman spectra. Figure 1b shows a Raman spectrum of NDC which exhibited the typical two broad bands at 1344 and 1588 cm^−1^ for the disordered (D) band and graphitic (G) band. The G band is predominantly observed for the sp^2^ carbon domains which attributed to the first-order scattering of the stretching vibration mode (E_2g_)^29^. The high G band intensity pronounces that the compound has a high crystallinity. Conversely, the D band is corresponds to structural disorder and defects which breaking the sp^2^ symmetry of carbon^30^. These defects and disorder was mainly produced by the presence of hetero atoms, which collapses the ordered crystallization of carbon to the hexagonal lattice^31^. The crystallinity of NDC was inferred from the relative intensities ratio of D and G bands (I_D_/I_G_). It exhibited that the I_D_/I_G_ is 0.81, which revealed the NDC was crystallized in highly oriented hexagonal carbon lattice^32^. The surface morphology was probed in the aspect of grain boundary and local crystallinity by transmission electron microscopy (TEM). Commonly, the flame synthesis method furnished a particle like surface morphology for the synthesized carbon nanomaterial^33^. Incidentally, the NDC also attains the similar particle like shape with apparent grain boundaries. But, in many places those particles are extended its grain boundaries and grown as sheets (Fig. 1c). In addition, the N-dopant was primarily analyzed by the energy dispersive x-ray analysis (EDAX), which results that the presence of C, O and N with the ratio of 88.0, 8.6 and 3.4%, respectively (Fig. 1f). X-ray photoelectron spectroscopy (XPS) was further used to confirm the N-dopant. Figure 1d displays the XPS high resolution N1s spectra of NDC, which showed the broad peak centered at 399.5 eV. This peak was de-convoluted into three different peaks which revealed the binding energy of 399.42, 398.79, and 400.1 eV. The observed peaks are often attributed to the pyrrolic, pyridinic, and graphitic nitrogen environment^27^. This result confirmed that the N atom was successfully doped in the carbon lattice.

### Electrochemical properties of modified GCEs

**Figure 2:**
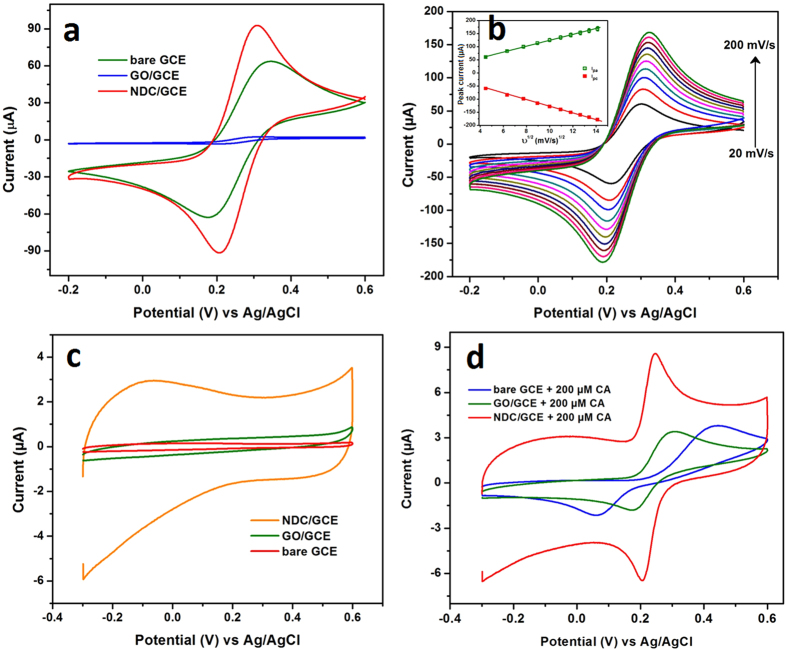
CV responses of bare GCE, GO/GCE and NDC/GCE in 0.1 M KCl containing 5 mM [Fe(CN)_6_]^3−/4−^ (**a**) and the different scan rate from 20–200 mV/s (**b**), inset shows the corresponding plot of square root of scan rate vs peak current. The CVs of bare GCE, GO/GCE and NDC/GCE in 0.05 M PB solution, in the absence (**c**) and presence of 200 μM CA (**d**).

The electrochemical behavior of the bare GCE, GO/GCE and NDC/GCE was studied in the 0.1 M KCl containing 5 mM [Fe(CN)_6_]^3−/4−^ redox probe. Figure 2a shows the CV responses of bare GCE, GO/GCE and NDC/GCE in [Fe(CN)_6_]^3−/4−^, which exhibited that the pair of well-defined redox peaks for the redox reaction of [Fe(CN)_6_]^3−/4−^. However, the NDC/GCE revealed a higher redox peak current and smaller peak-to-peak potential separation compared to the bare GCE and GO/GCE. Furthermore, the redox peak current of the GO/GCE is significantly lower than that of bare GCE and NDC/GCE. It is obvious to discriminate that the more oxygen functionalities of GO influences the insulating behavior. Hence, it blocks the diffusion of [Fe(CN)6]^3−/4−^ and also increased the internal resistance at the electrode interface^34^. The peak-to-peak potential separations (ΔEp) of bare GCE, GO/GCE and NDC/GCE are showed as 170, 120 and 99 mV respectively. Among them, the NDC/GCE was revealed the low ΔEp, because of the highly oriented hetero atom doped carbon lattice. In addition, the NDC/GCE exhibited the ratio of anodic to cathodic peak current (I_pa_/I_pc_) is 0.75 (1**≈** reversible) which is confirmed that the redox reaction of [Fe(CN)_6_]^3−/4−^ was quasi-reversible at NDC/GCE. To find out the effective electrochemical active surface area of NDC/GCE, the redox properties of NDC/GCE was studied in [Fe(CN)_6_]^3−/4−^ at various scan rates ranging from 20 to 200 mV/s (Fig. 2b). The electro active surface area of the modified electrode was calculated by the Randles– Sevcik equation^35^:


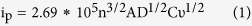


where, i_p_ is the peak current, D is the diffusion coefficient (cm^2^ s^−1^), C is the concentration of the [Fe(CN)_6_]^3−/4−^ molecules (mol L^−1^), A is the electrochemical active area (cm^2^), n is the number of electron transfer and 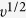 is the scan rate (V s^−1^). From the slopes of the I_pa_ versus 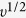 (Fig. 2b, inset) the electro active surface areas were calculated to be 0.038, 0.003, and 0.045 cm^2^ for the bare GCE, GO/GCE and NDC/GCE respectively. These results indicated that the NDC/GCE has a high electroactive surface area, due to the highly oriented carbon lattices enriched with the N atoms.

Electrochemical impedance spectroscopy (EIS) is an efficient technique to intrigue the electron transfer properties of surface modified GCEs. Figure 1e shows the EIS spectrum of the bare GCE, GO/GCE and NDC/GCE recorded in 0.1 M KCl containing 5 mM [Fe(CN)_6_]^3−/4−^ redox probe. The typical Nyquist plot of the NDC/GCE exhibited the semicircle with a charge transfer resistance (*R*_ct_) of 0.08 kΩ, this is quite smaller when compared with bare GCE and GO/GCE (Fig. 1e, inset). However, the GO/GCE revealed higher *R*_ct_ value of 13.1 kΩ which implies that the GO/GCE has high resistance due to more oxygen functionalities. It can be noted that these oxygen functional groups blocked the diffusion of redox probe and also created more resistance at electrode-electrolyte interface. The EIS results of all modified GCEs were consistent with the CV responses of redox probe (Fig. 2a). These results are indicated that the NDC/GCE has the excellent electrochemical properties than that of bare GCE and GO/GCE.

### Electrochemical behavior of CA

The electrocatalytic oxidation of CA on the bare GCE, GO/GCE and NDC/GCE was primarily assessed by cyclic voltammetry. Figure 2c shows the CV responses of modified GCEs, which are recorded in 0.05 M phosphate buffer (PB) solution at a scan rate of 50 mV/s. As shown in Fig. 2c, no obvious redox peaks were appeared for the modified GCEs in the absence CA in PB solution. Apart from that, a larger background current was appeared for the NDC/GCE, which is due to the interaction of electrolyte ions with the interlayer of graphitic NDC surface. It enhances the electrochemical double layer capacitance of the NDC by the K^+^ intercalation. However, bare GCE and GO/GCE exhibited lower background current due to the poor interaction with electrolyte ions. A pair of well-defined quasi-reversible redox peaks appeared for the modified and bare GCE at various peak potential for the addition of 200 μM CA (Fig. 2d).

The observed redox peaks are attributed to the formation of o-quinone, these redox reactions of CA were followed the two electron transfer process^36^. In contrast with the GO/GCE and bare GCE, the NDC/GCE showed the well-shaped and sharp redox peak with high current. Moreover, the oxidation/reduction peak potential of CA was lower than that of bare GCE and GO/GCE. This high electrocatalytic oxidation of CA at NDC/GCE indicated that the NDC has more electroactive functional groups. These functional groups are so-called the pyridinic and pyrrolic groups which are embedded in the highly oriented NDC lattice^27^. However, the GO/GCE and bare GCE showed a relatively weak oxidation peak at 0.304 V and 0.443 V respectively, which are somewhat high potentials than NDC/GCE. Moreover, the redox peak current also approximately two times lower than that of NDC/GCE. In addition, the peak-to-peak separations for the CA oxidation were obtained as 40, 132 and 385 mV for NDC/GCE, GO/GCE and bare GCE. These results evidenced that the NDC/GCE exhibits the excellent electrocatalytic activity towards the oxidation of CA. These superior electrocatalytic activities of NDC were ascribed from the electroactive functional groups of hetero atom enriched carbon lattices.

### Effect of scan rate and pH

**Figure 3:**
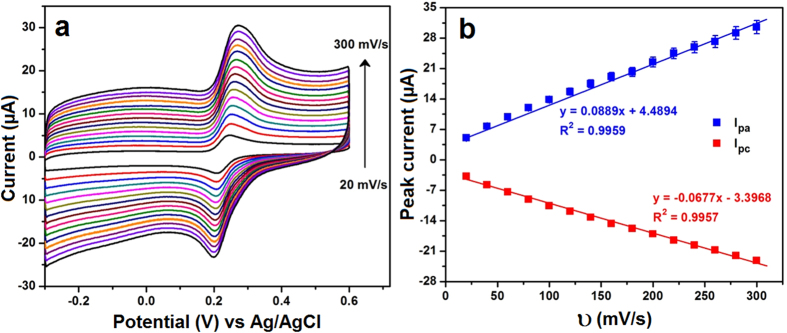
The CV curves of NDC/GCE in 0.05 M PB solution containing 200 μM CA for the different scan rates ranging from 20 to 300 mV/s (**a**) and the corresponding plot of redox peak current vs. scan rate in (**b**).

The electrocatalytic behavior of CA was further evaluated based on the effect of scan rate. Figure 3a displayed the CV responses of the NDC/GCE recorded in 0.05 M PB solution containing 200 μM CA for the various scan rates from 20 to 300 mV/s. The oxidation/reduction peak currents of the CA increased with increasing the scan rates, however, the consequent peak potentials were slightly shifted. This relocated potential was influenced by the size of the diffusion layer which depends on the scan rate. At a lower scan rates, the thickness of the diffusion layer is high and it has been grown much further from the electrode surface. In contrast, the thickness of diffusion layer is considerably low at high scan rates. As a result, the altering flux is drastically lower at the electrode surface when sweeping the potential at lower scan rates, hence, the peak potential was shifted. In addition, the peak current of CA oxidation was plotted against the scan rate and shown in Fig. 3b. The observed plot indicated that the CA oxidation had a linearity with the linear regression equation of Ipa (μA) = 0.0889 × 10^−6^ A/mVs^−1^ + 4.489 μA (R^2^ = 0.9959) and Ipc (μA) = 0.0677 × 10^−6^ A/mVs^−1^ − 3.396 μA (R^2^ = 0.9957). This study indicated that the electrocatalytic oxidation of CA at NDC/GCE was controlled by the adsorption controlled process^22^.

**Figure 4:**
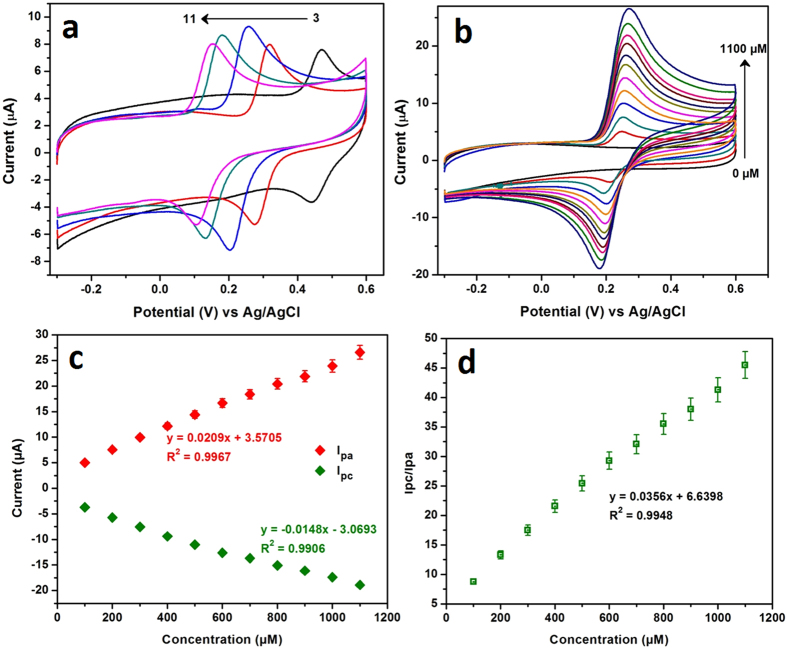
The CV responses of the oxidation of CA at NDC/GCE in various pHs ranging from 3.0 to 11.0 (**a**). The CV curves of NDC/GCE in various concentrations of CA ranging from 0 to 1100 μM (**b**) and the corresponding plot of redox peak current vs. concentration of CA (**c**). The ratio of the redox peak currents of CA is plotted against the concentration of CA, which follows the linearity with the slope of 0.0356 (**d**).

The pH of the electrolyte significantly influenced the electrochemical behavior of CA, hence, the oxidation of CA was studied by CV in the various pH solutions containing 200 μM CA at a scan rate of 50 mV/s. Figure 4a, shows the CV responses of the CA oxidation at NDC/GCE for the different pH ranging from 3.0 to 11.0. It can be seen that, the peak potentials of the CA oxidation curves were shifted to the more negative potential when increase the pHs^21^. The anodic peak potentials (E_pa_) did not follow the linear relationship against the pHs. The NDC/GCE exhibited an almost similar oxidation peak current for the pHs, 3.0, 5.0, 9.0 and 11.0, however, slightly enhanced for pH 7.0. This investigation resulted that the CA oxidation at various pHs didn’t showed any appreciable linearity over the pHs. This is because of the surface confined adsorption of the CA at NDC surface.

### Effect of concentration

The electrochemical behavior of CA at NDC/GCE was further investigated for the various concentration of CA in 0.05 M PB solution (pH = 7.0) at a scan rate 50 mV/s. It can be noted that from Fig. 4b, a sharp response was arisen for the addition of 100 μM of CA whereas no obvious redox peak was observed for the absence of CA. The anodic and cathodic peak currents of the CA were linearly increased with increasing the concentrations of CA from 0 to 1100 μM. The redox peak currents of the CA oxidation/reduction were plotted against the concentrations of CA (Fig. 4c) which followed the linearity with linear regression equations of Ipa (μA) = 0.0209 μA/μM + 3.57 μA (R^2^ = 0.9967) and Ipc (μA) = −0.0148 μA/μM − 3.06 μA (R^2^ = 0.9906). In addition, the ratio of the redox peak currents (Ipc/Ipa) was evaluated to assess the reversibility of the CA oxidation at NDC/GCE. Figure 4d shows the plot of concentration vs Ipa/Ipc, where the linearity was observed with the slope of 0.0356 μM^−1^. This slope value indicated that the reversibility of CA oxidation was 0.0356 times reduced over the concentrations. At lower concentrations the CA oxidation is almost reversible, however, at higher concentrations the CA oxidation was quasi reversible. This reversibility was influenced by the micro pores of the NDC, which leads to the surface confined CA adsorption. As discussed in scan rate, the electrochemistry of the CA oxidation at NDC was controlled by adsorption process. Hence, there is a minimal possibility to diffuse the CA at NDC surface. As a result, the reversibility of CA oxidation was affected at higher concentrations.

### Determination of CA on NDC/GCE

**Figure 5:**
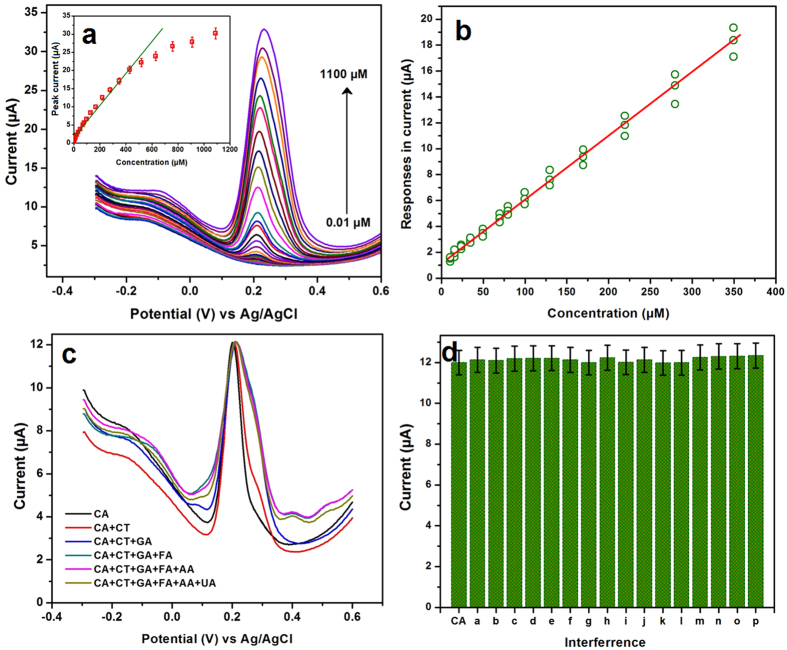
The DPV responses of NDC/GCE in various concentrations of CA from 0.01 to 1100 μM in 0.05 M PB solution (**a**) and the corresponding calibration plot shown in inset. (**b**) Calibration data for the determination of CA by the DPV method for three duplicated measurements with *n* standards. (**c**) The DPV responses of CA oxidation in presence of potential interference, such as catechol (CT), gallic acid (GA), ferulic acid (FA), ascorbic acid (AA) and uric acid (UA) (**b**). (**d**) The DPV interference signals of CT (*a*), GA (*b*), FA (*c*), AA (*d*), UA (*e*), Na^+^ (*f*), K^+^ (*g*), Mg^2+^ (*h*), Ni^2+^ (*i*), Cu^2+^ (*j*), Cl^−^ (*k*), NO_3_^−^ (*l*), SO_4_^2−^ (*m*), glucose (*n*), DA (*o*) and H_2_O_2_ (*p*) where CA is the standard for comparison.

Differential pulse voltammetry (DPV) is more sensitive tool to quantify the concentrations of CA, hence, the DPV was used to determine the CA. Figure 5a shows the DPV responses of CA oxidation curves in 0.05 M PB (pH = 7) solution containing various concentrations of CA from 0.01 to 1100 μM. A sharp oxidation response was appeared for the addition of 0.01 μM CA, then the oxidation peak currents were consecutively increased with increasing the concentrations. The observed oxidation peak current of the CA oxidation was increased with increasing the concentrations ranging from 0.01 to 440 μM with the R^2^ = 0.9876 (Fig. 5a, inset). However, it is not followed the considerable linearity for the concentration and response signal. Hence, the linearity of the calibration plot was analyzed by the F-test. The three set of measurements (p) was carried out for the 14 standards (n). All the replicated responses for the duplicated experiments are shown in Fig. 5b. It can be seen that, the standard deviation of analyte responses were increased when increasing the concentration. This type of expression is called a heteroscedastic case. Prior to calculate the F value, firstly, the linear regression is needed to fit by weighted least squares. Herein, the weights are calculated by w_*i*_ = σ_*i*_^−2^ where σ_*i*_ is the standard deviation of the voltammetric responses at the concentration c_*i*_. From the calibration data, we have seen that the response was very low at 0 concentration therefore it was replaced by possible value. Finally, according to K. Danzer and L. A. Currie, the experimental F value for the α = 0.05, F_12,28_ is calculated to be 2.12 which is more close to the tabulated critical F value^37^,^38^. This result exhibited that there is no significant lack of fit. From this data, the lowest detection limit (LOD) and the limit of quantification (LOQ) were calculated by the modern IUPAC recommendation based on the type I error (false positive, possibility: mostly <1%) and type II error (false negative, possibility ∼50%)^39^. The calculated LOD and LOQ are to be 0.0024 μM and 0.004 μM. This superior electrocatalytic performance of the NDC/GCE-CA sensor was compared with the previously reported sensors and given in Table 1. Among the developed CA sensors (from Table 1), the NDC/GCE-CA sensor exhibited the wide linear concentration range and the lowest detection limit.

### Effect of electrolyte

**Figure 6:**
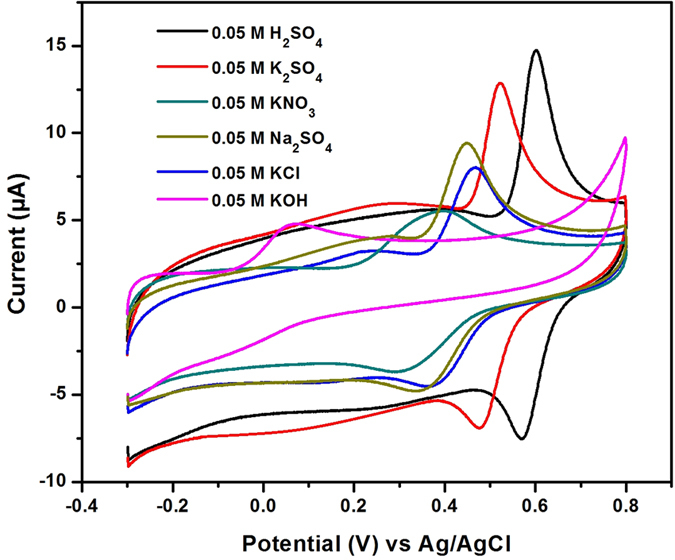
The CV responses of CA oxidation at NDC/GCE for the 200 μM CA recorded in 0.05 M solution of various electrolytes such as KNO_3_, KCl, K_2_SO_4_, KOH, Na_2_SO_4_, and H_2_SO_4_ at the scan rate of 50 mV/s.

To examine the electrochemical robustness of developed sensor matrix for the detection of CA, the influence of supporting electrolytes were analyzed. Figure 6 shows the CV responses of NDC/GCE for 200 μM CA recorded in 0.05 M solution of various electrolytes such as KNO_3_, KCl, K_2_SO_4_, KOH, Na_2_SO_4_, and H_2_SO_4_ at the scan rate of 50 mV/s. As said in pH study, the electrocatalytic responses are varying for different pHs as well as the peak shape also changed. Indeed, the positive or negative species of the electrolyte ions maybe interfere with the electrocatalytic activity. As shown in Fig. 6, a better redox behavior was observed for CA on NDC/GCE while using the H_2_SO_4_ as an electrolyte. However, the strong acidic medium is not recommended always for the practical applications due to the corrosion. This study revealed the understanding of electrochemical robustness of the developed sensor matrix. Therefore, the developed sensor matrix can be applicable for the versatile electrolyte system although it was highly active in PB solution.

### Selectivity, reproducibility and stability

**Figure 7:**
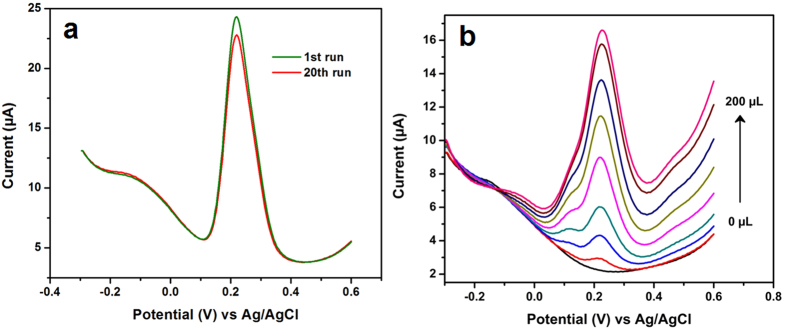
The DPV responses of CA oxidation at NDC/GCE for consecutive 20 measurements (**a**) and the determination CA in red wine sample (**b**).

Selectivity is a significant parameter in the development of sensors, most of the similar structural compounds or some common ions are effectively interfered with the determination of CA. Hence, the interference study was evaluated for the developed CA sensor with potential interfering ions. Figure 5c shows the DPV responses of CA with 10-fold excess of interfering ions such as catechol (CT), gallic acid (GA), ferulic acid (FA), ascorbic acid (AA) and uric acid (UA). This study indicated that no interference current was observed for the developed CA sensor with those interfering compounds. Furthermore, the selectivity was evaluated in 200 fold excess of Na^+^, K^+^, Mg^2+^, Ni^2+^, Cu^2+^, Cl^−^, NO_3_^−^, SO_4_^2−^, glucose, dopamine (DA) and hydrogen peroxide (H_2_O_2_). The interference response signal was not more than 5% (Fig. 5d), therefore, the NDC/GCE sensor can be applicable to the real time sensor for the determination of CA. The stability and reproducibility of the developed CA sensor were investigated by the DPV. Figure 7a depicts the DPV responses of CA oxidation in 0.05 M PB solution containing 100 μM CA for 20 consecutive measurements. It can be seen that, a small variation was observed in the oxidation current of the CA for the 20 measurements with the RSD of 1.88%. In addition, the oxidation peak current was retained about 93% from its initial current. This result endorsed that the developed sensor has the good stability and acceptable repeatability for the CA detection. In addition, the developed CA sensor electrode was scrutinized for the consecutive weeks to assess the storage ability. It was showed the retention peak current about 93.49% after 6 weeks for the 20 measurements, which indicates that the developed NDC/GCE sensor has good storage stability. These studies resulted that the NDC/GCE has good selectivity, stability and reproducibility to the determination of CA.

### Real sample analysis

Practicability of the developed CA sensor was evaluated in red wine towards the determination of CA. Five samples of various red wines are used for this experiment and each sample was tested by three experiments (n = 3). Here, the experiment was performed by directly adding the raw wine in 0.05 M PB solution and determined the concentrations of CA. Figure 7b shows the DPV responses of CA determination in 0.05 M PB solution by adding the various amounts of red wine sample ranging from 5 to 200 μL. By comparing the peak currents of CA oxidation with the known calibration plot (Fig. 5a), the concentration of CA in red wine was calculated to be 98.4 μM for sample A with the RSD of 2.1%. The same procedure was followed for other samples and the results were given in Table 2.

## Conclusions

A selective and sensitive electrochemical CA sensor was demonstrated based on the NDC. The nitrogen was successfully doped in the highly oriented hexagonal carbon lattice which was confirmed by the various physical characterizations. The electrochemical properties of the CA oxidation were investigated in various concentration of CA and portrayed its performance. In contrast with the bare GCE and GO/GCE, NDC/GCE exhibited an excellent performance towards the determination of CA. This high performance was ascribed from the pyridinic and pyrrolic functionalities of NDC. The NDC/GCE was used for the determination of CA, it revealed the wide linear concentration range from 0.01 to 350 μM with the lowest detection limit of 0.0024 μM and attained the limit of quantization of 0.004 μM. The developed sensor was achieved the good selectivity, stability, sensitivity and reproducibility, furthermore, it was successfully applied to the determination of CA in wines. These factors consistently make the NDC as a promising sensor material for the electrochemical determination of CA. In addition, it can be applicable to the real time sensing of CA in pharmacological studies and quality control analysis.

## Experimental

### Materials

The pyrrole (≥98%), isopropyl alcohol (≥99.7%), caffeic acid (≥98%), catechol (≥99%), gallic acid (97.5–102.5%), ferulic acid (≥99%), ascorbic acid (≥99%) and uric acid (≥99%) were obtained from Sigma-Aldrich and used without further purification. All other chemical & reagents were analytical grade and used without further purification. The potassium dihydrogen phosphate (98%) and potassium monohydrogen phosphate (95%) salts are purchased from alfa aesar and all other chemicals are analytical grade. All required solution prepared by using deionized water (DI) water.

### Synthesis of Nitrogen doped carbon

The NDC was synthesized by our previously reported flame synthesis method with some modifications^27^. The pyrrole and isopropyl alcohol was taken in the ratio of 93:7 and dispersed well and ultrasonicated for an hour. After that, the mixture was taken in small beaker and ignited on the top of the beaker. It burns with sooty flame and then the resultant soot was carefully deposited on a glass plate. The deposited carbon was collected by scratching and directly used for the further experiments without purification.

### Fabrication of modified electrodes

A 40 mg of NDC was dispersed in 5 mL of DI water and ultrasonicated for 15 min. Then allowed to form a homogeneous solution, about 8 μL of that solution was drop casted on the well-polished GCE and dried at room temperature. This NDC modified electrode was used for the further electrochemical measurements.

### Characterization of NDC

The structural behavior of the NDC was analyzed by X-ray diffraction pattern analysis (XRD), XPERT-PRO (PANalytical B.V., The Netherlands) with CuKα radiation (λ = 1.5406 Å). The defects and disorder of NDC was performed by Raman spectra (NT-MDT, NTEGRA SPECTRA). The surface morphology was probed by Transmission electron microscope (TEM- TECNAI G^2^). The electrocatalytic behavior and the determination of CA were performed by cyclic voltammetry (CV) and differential pulse voltammetry (DPV), CHI 405a and CHI 900 (CH Instruments, USA). The DPV studies were recorded by applying the potential window from −0.3 to 0.6 V with the optimized pulse amplitude (0.05 V) and pulse width (0.05 s). A conventional three electrode system has been used for the electrocatalytic studies where the modified GCE is working electrode (0.07 cm^2^), platinum wire is an auxiliary electrode and Ag/AgCl-3 M KCl is used as a reference electrode.

## Additional Information

**How to cite this article:** Karikalan, N. *et al*. A voltammetric determination of caffeic acid in red wines based on the nitrogen doped carbon modified glassy carbon electrode. *Sci. Rep.* **7**, 45924; doi: 10.1038/srep45924 (2017).

**Publisher's note:** Springer Nature remains neutral with regard to jurisdictional claims in published maps and institutional affiliations.

## Figures and Tables

**Table 1:** Comparison of the developed NDC/GCE-CA sensor with other reported CA sensors.

Modified electrodes	Method	Linear range (μM)	Limit of detection (μM)	Ref
Molecularly imprinted siloxanes	DPV	0.500–60.0	0.15	13
RGO/PDA	DPV	0.005–450.5	0.0012	40
Electrochemically reduced graphene oxide–Nafion	SWAdSV	0.1–10	0.09	22
Laccase-MWCNT-chitosan/Au	Amperometric	0.7–10	0.15	19
Nafion/Tyre/Sonogel-Carbon	Amperometric	0.08–2	0.06	41
Gold nanoparticles (AuNPs) and graphene nanosheet (GN) modified glassy carbon electrode	CV	0.5–50	0.05	20
Graphene oxide nanosheets	DPV	0.5–100	0.0011	42
Glassy polymeric carbon	DPV	96.5–0.1	0.29	17
Activated GCE	CV	0.1–1	0.068	43
Glassy carbon electrode	DPV	10–120	0.1	44
Nitrogen doped carbon/GCE	DPV	0.01–350	0.0024	This work

**Table 2:** Determination of CA in red wines by NDC/GCE-CA sensor.

Red wine sample	Found (μM)	RSD (%)
A	98.4	2.1
B	72.6	2.3
C	81.5	2.0
D	78.6	2.1
E	65.4	2.5
